# Surface modification of a POSS-nanocomposite material to enhance cellular integration of a synthetic bioscaffold

**DOI:** 10.1016/j.biomaterials.2016.01.005

**Published:** 2016-03

**Authors:** Claire Crowley, Poramate Klanrit, Colin R. Butler, Aikaterini Varanou, Manuela Platé, Robert E. Hynds, Rachel C. Chambers, Alexander M. Seifalian, Martin A. Birchall, Sam M. Janes

**Affiliations:** a Lungs for Living Research Centre, UCL Respiratory, University College London, London, UK; b UCL Centre of Nanotechnology and Regenerative Medicine, Division of Surgery and Interventional Science, Royal Free London NHS Foundation Trust Hospital and University College London, London, UK; c Centre for Inflammation and Tissue Repair, UCL Respiratory, University College London, London, UK; d UCL Ear Institute, Royal National Throat Nose and Ear Hospital and University College London, London, UK

**Keywords:** Tissue engineering, Biocompatible materials, Porosity, Nanocomposites, Re-epithelialization, Trachea

## Abstract

Polyhedral oligomeric silsesquioxane poly(carbonate-urea) urethane (POSS-PCU) is a versatile nanocomposite biomaterial with growing applications as a bioscaffold for tissue engineering. Integration of synthetic implants with host tissue can be problematic but could be improved by topographical modifications. We describe optimization of POSS-PCU by dispersion of porogens (sodium bicarbonate (NaHCO_3_), sodium chloride (NaCl) and sucrose) onto the material surface, with the principle aim of increasing surface porosity, thus providing additional opportunities for improved cellular and vascular ingrowth. We assess the effect of the porogens on the material's mechanical strength, surface chemistry, wettability and cytocompatibilty. Surface porosity was characterized by scanning electron microscopy (SEM). There was no alteration in surface chemistry and wettability and only modest changes in mechanical properties were detected. The size of porogens correlated well with the porosity of the construct produced and larger porogens improved interconnectivity of spaces within constructs. Using primary human bronchial epithelial cells (HBECs) we demonstrate moderate *in vitro* cytocompatibility for all surface modifications; however, larger pores resulted in cellular aggregation. These cells were able to differentiate on POSS-PCU scaffolds. Implantation of the scaffold *in* v*ivo* demonstrated that larger pore sizes favor cellular integration and vascular ingrowth. These experiments demonstrate that surface modification with large porogens can improve POSS-PCU nanocomposite scaffold integration and suggest the need to strike a balance between the non-porous surfaces required for epithelial coverage and the porous structure required for integration and vascularization of synthetic scaffolds in future construct design.

## 1 Introduction

Tissue engineering is evolving with a plethora of candidate scaffold materials [1]. Clinically, a number of organs have now been transplanted using artificial scaffolds including bladder, esophagus and trachea [2], [3]. The optimal scaffold material in each setting remains undetermined with debate largely focused on whether biological (e.g. decellularized) or synthetic material is most appropriate [4], [5]. Whilst the former is often considered superior as it mimics native tissue, it is wholly reliant on procuring donor tissue [6]. Synthetic biomaterials have the potential to overcome this problem and advances in manufacturing techniques, including surface modifications, mean synthetics hold increasing promise [7], [8].

Polyhedral-oligomeric silsesquioxane (POSS), (RSiO3/2) incorporated into poly(carbonate-urea) urethane (PCU) is one such synthetic material developed by our group [9]. As a nanocomposite material, it has improved structural properties, as well as enhanced biostability, gas barrier, thermal stability, elastic modulus and mechanical properties compared to other conventional microcomposites [10], [11]. This is because its smaller particle size leads to an increase surface to volume ratio, permitting a higher number of reactions to occur on the particle surface [12]. Extensive testing has confirmed this material meets international standards (ISO 10993) for biocompatibility, supporting various cell types [13], [14] and a number of tissues/organs have now been created using POSS-PCU, including trachea [15], facial organs [16], [17], lacrimal ducts [18] and vascular bypass grafts [19], [20], [21]. Transcatheter heart valves and coronary artery bypass grafts using POSS-PCU are undergoing pre-clinical and clinical trials [20], [22].

Organs that form tubular conduits, such as trachea and esophagus, are a challenge for bioengineers as they represent an interface with the outside environment, making barrier function essential [23], [24], [25], [26]. Current use of POSS-PCU involves a semi-closed pore design to help maintain this surface integrity. However, such an approach could limit delivery of nutrients and metabolites within the implanted material. Adequate porosity of the scaffold has been shown to improve outcomes in tissue-engineered bone and cartilage where it is essential to permit neoangiogenesis [27]. As such, future scaffold designs should incorporate a degree of permeability to encourage tissue engraftment and angiogenesis [28]. We have attempted to address this challenge by using a variety of porogens in the manufacture process of POSS-PCU scaffolds. Permeability, surface and biomechanical properties were characterized following porogen modification. Cytocompatibility was demonstrated by seeding primary human epithelial cells sourced from large airways at varying seeding concentrations and examining cell viability, proliferation and differentiation. The integration capacity of treated and non-treated three-dimensional (3D) sheets of the POSS-PCU foam-form elastomer was determined by implantation *in vivo*.

## 2 Materials and methods

### 2.1 POSS-PCU nanocomposite polymer synthesis and fabrication

POSS-PCU was synthesized using a previously described technique. Briefly, *trans*-cyclohexanechloroydrinisobutyl-silsesquioxane (Hybrid Plastics, USA) and polycarbonate polyol (2000 MW) were mixed in a reaction flask, heated to 115 °C to dissolve the POSS nanocage into the polyol and then cooled to 60 °C. Flake 4,4′-methylene bis(phenyl isocyanate) was added to the polyol blend and reacted under nitrogen for 120 min at 65 °C to form a pre-polymer. Dimethylacetamide (DMAC) was added slowly to the pre-polymer to form a solution and was then cooled to 35 °C. Chain extension of the pre-polymer was carried out by the dropwise addition of ethylenediamine in dimethylacetamide to form a solution of POSS-modified polycarbonate urea-urethane in dimethylacetamide. All chemicals and reagents were purchased from Sigma–Aldrich Ltd., Gillingham, UK.

The polymer was processed into a foam elastomer by coagulating the polymer in water, leaving it for three days and changing the water three times daily thereafter. Where indicated, the top layer of the polymer was altered by dusting various porogen types onto the surface. Three different porogens were used, NaHCO_3,_ NaCl and sucrose. Each porogen type was sprinkled onto a polymer sheet (surface area of 64 cm^2^) and the dish was immersed in distilled water. To determine the average grain size of each porogen type, the porogens were imaged with scanning electron microscopy using a Jeol 7401 FEGSEM microscope. The porogens were sprinkled onto aluminum stubs using sticky carbon tabs and coated with a thin layer of Au/Pd (approximately 2 nm thick) using a Gatan ion beam coater. The maximum diameter of each grain was then measured using the measuring tool on the Joel software and the mean was calculated using GraphPad prism.

### 2.2 Characterization of the 3D construct

#### 2.2.1 Mechanical properties

The tensile properties of each sample were tested using an Instron 5565 tester (Instron Ltd, Bucks, UK). Each sample was cut into five dumbbell shapes using a template cutter and the sample thickness was recorded. The tensile stress versus strain properties were assessed according to ISO 37 using dumbbell-shaped specimen type 3 (16 mm gauge length and 4 mm width) at a displacement rate of 200 mm min^−1^. Young's modulus was also calculated using Bluehill software as the slope of the straight-line portion between 0 and 5 mm.

#### 2.2.2 Surface chemical properties using FTIR

The surface chemical composition of six of each sample type was characterized. Fourier Transform Infra-Red spectra were recorded on a Jasco FT-IR 4200 Spectrometer equipped with a diamond attenuated total reflectance accessory (Diamond MIRacle ATR, Pike Technologies, USA). An average of 20 scans at 4 cm^−1^ resolution over a range of 600 cm^−1^ to 4000 cm^−1^ wavenumbers were performed to produce the spectra.

#### 2.2.3 Surface hydrophobicity using captive bubble contact angle

Water contact angle analysis was used to determine the hydrophilicity/hydrophobicity of the material surface. The captive bubble method for assessment was applied as this method does not require the refabrication of samples, which can disturb prospective nuances of the surface. The angle between an air bubble and the POSS-PCU samples immersed in water was measured (Supplementary Figure 1). Samples (n = 6) were analyzed using a KRÜSS DSA 100, Drop Shape Analyzer (KRÜSS GmbH, Hamburg, Germany). The contact angles on both sides of the bubble were calculated and averaged producing the overall theta value. Samples were autoclaved prior to testing and analyzed with the treated side in contact with the air bubble.

#### 2.2.4 Permeability of 3D POSS-PCU scaffolds

The permeability of each scaffold type (n = 3) was determined using a custom-made apparatus designed to measure the flow rate of water through a 1 cm^2^ aperture containing the sample, with a controlled pressure. The permeability was considered the degree of flow through a sample (ml/min).

#### 2.2.5 Surface morphology using scanning electron microscopy (SEM)

Scanning electron microscopy (SEM) images of the apical and basal surfaces of the scaffold as well as scaffold cross sections were taken. This was performed in the same way for seeded, unseeded and implanted scaffolds. Samples were fixed in 2.5% glutaraldehyde in PBS. Following fixation, scaffolds were washed in 0.1 M phosphate buffer (pH 7.4) followed by distilled water, dehydrated in a graded ethanol-water series to 100% ethanol and critical point dried using carbon dioxide. The samples were then mounted on aluminum stubs using sticky carbon tabs, so that the surfaces of interest were presented to the beam. Specimens were coated with a thin layer of Au/Pd (approximately 2 nm thick) using a Gatan ion beam coater and viewed using a Jeol 7401 FEGSEM. To quantify the mean surface pore size of each scaffold type, Volocity software (PerkinElmer) was used to measure the maximum diameter of each pore and the mean was calculated using GraphPad Prism software v6.

### 2.3 Cytocompatibility experiments

#### 2.3.1 Cell culture

Primary human bronchial epithelial cells (HBECs) were obtained from regions of normal airway from patients undergoing lobectomy (Research Ethics Committee 06/Q0505/12) or purchased from Sciencell (California, USA).

Airway sections were cut macroscopically under sterile conditions into 5 mm^2^ pieces and incubated in a solution of 0.15% (w/v) pronase (Roche, Basel, Switzerland) in Dulbecco's modified Eagle's medium (DMEM, PAA Laboratories GmbH, Pasching, Austria) at 4 °C overnight with agitation. This reaction was neutralized using 20% fetal bovine serum (v/v; FBS; Life technologies, Carlsbad, CA, USA). Primary cells were pelleted and resuspended in bronchial epithelial growth medium (BEGM; CC-3170, Lonza, Switzerland) and seeded at 1 × 10^6^ cells/25 cm^2^ in tissue culture flasks (Nunc International, Penfield, NY, USA). Cells were incubated at 37 °C with 5% CO_2_ and allowed to adhere for 24 h prior to medium change. Medium changes were performed every 48–72 h. Cells were expanded to passage 3 for all experiments.

#### 2.3.2 Cell seeding and cell viability on scaffolds

POSS-PCU sheets were cut into circular discs to fit 96-well plates using a punch biopsy for both dusted and non-dusted samples (n = 3) and the discs were washed in distilled water 3 times a day for 2 days in order to remove residual salt. The discs were sterilized by autoclaving in distilled water at 120 °C for 15 min and subsequently stored at 4 °C until use [29]. The discs were then placed in 96-well plates under sterile conditions in BEGM overnight at 37 °C with 5% CO_2_. The next day the medium was removed by aspiration and HBECs were seeded onto the scaffolds in BEGM at different seeding densities (1, 2.5, 5 and 10) x 10^5^ cells/cm^2^. Each seeding density was repeated in triplicate on each scaffold type. Experiments were repeated with cells from 3 different donors. Scaffolds without cells were maintained in the same conditions as a negative control. As a positive control, cells were seeded in wells coated with 50 μg/cm^2^ collagen IV (Sigma–Aldrich, Gillingham, UK). Cell viability and proliferation were assessed using an alamarBlue assay (Invitrogen, Carlsbad, CA, USA) performed according to manufacturer's instructions. One day post seeding, scaffolds were transferred to a new 96-well plate to avoid false positive readings from cells adhering to the tissue culture plastic rather than the scaffold. Readings were taken at day 1, 2, 3, 4 and 5.

#### 2.3.3 Immunocytochemistry, immunohistochemistry and immunofluorescence

HBECs grown in chamber slides (BD Biosciences, Franklin Lakes, NJ, USA) were fixed in 4% (w/v) paraformaldehyde (PFA, Sigma–Aldrich, Gillingham, UK) in phosphate buffered saline (PBS, PAA Laboratories GmbH, Pasching, Austria) for 10 min at room temperature and kept in PBS for subsequent immunofluorescence staining. After 2 or 5 days of submerged culture, POSS-PCU scaffolds were removed from plates and washed three times in PBS to remove residual medium. The scaffolds were PFA-fixed for 30 min at room temperature and washed three times in PBS before immunofluorescence staining. Samples retrieved from *in vivo* studies were paraffin-embedded and sectioned for haematoxylin and eosin (H&E), Masson's trichrome [22] and Periodic Acid Schiff (PAS) staining. Slides were digitally imaged using a NanoZoomer 2.0-HT system and NanoZoomer Digital Pathology software (Hamamatsu Photonics, Japan) at 40× magnification.

For immunofluorescence, paraffin sections were dewaxed and underwent antigen retrieval with citrate buffer. All samples were blocked using 1% bovine serum albumin in PBS (w/v; BSA; Sigma–Aldrich, Gillingham, UK) at room temperature, followed by incubation in primary antibody diluted in blocking solution plus 0.1% Triton X-100 (Sigma–Aldrich, Gillingham, UK) for 2 h at room temperature. Samples were washed three times with PBS and species-appropriate Alexa Fluor-conjugated secondary antibodies (Life Technologies, Paisley, UK), diluted 1:300 in block solution, were applied for 1 h at room temperature. DAPI, diluted in PBS, was applied to slides before a final three washes in PBS and coverslipping. Epithelial cells were identified by using primary antibodies against pan-cytokeratin (AE1/AE3 or ab9377; Abcam), cytokeratin 5 (CK5; mouse-anti-human, clone XM26 or rabbit-anti-human, polyclonal, Abcam), E-Cadherin (BD Biosciences, Oxford, UK), MUC5B (HPA008246; Sigma–Aldrich, Gillingham, UK), ACT (T6793; Sigma–Aldrich, Gillingham, UK), Ki67 (M7240; Dako) and Alexa Fluor 488-conjugated phalloidin to stain actin (Life Technologies, Paisley, UK). Endothelial cells were identified by an anti-CD31 antibody (ab28364; Abcam). Images were acquired using either a Zeiss LSM 500 confocal microscope (Carl Zeiss AG, Oberkochen, Germany) or an EVOS Imaging System (Life Technologies, Paisley, UK).

#### 2.3.4 Sterilization of POSS-PCU scaffolds

POSS-PCU scaffolds were sterilized either by autoclave at 120 °C, 15 psi above atmospheric pressure for 15 min or ethanol-cleansed by submerging scaffolds in 70% ethanol for 30 min followed by repeated PBS washing.

#### 2.3.5 Differentiation of HBECs on unmodified POSS-PCU scaffolds

HBECs (Sciencell, California, USA) were cultured in bronchial epithelial cell medium (BEpiCM, Sciencell, California, USA), trypsinized and viable cells counted using trypan blue staining (Life Technologies, Paisley, UK). Cells were seeded to be confluent at 24 h onto either Falcon 0.4 μm pore size polyethylene terephthalate (PET; Becton Dickinson Labware, Claix, France) as a positive control or POSS-PCU sheets clipped with Cellcrown (Sigma–Aldrich, Gillingham, UK) to create a POSS-PCU transwell insert. 500 μL of Pneumacult-ALI (STEMCELL Technologies, Cambridge, UK) was added into the lower chamber of each well while cells were seeded in a 200 μL volume in the upper compartment. After three days of submerged culture, scaffolds were air-lifted by adding 1 mL of Pneumacult-ALI to the basal chamber and removing media from the upper chamber to generate an air-liquid interface.

### 2.4 *In vivo* scaffold integration experiments

Scaffold discs were implanted either subcutaneously or into the dorsal muscle of C57BL/6 mice for 4 or 8 weeks. The study was approved by University College London ethics committee and performed under Project License PPL70/7504. Mice were obtained from an inbred colony, housed in individually ventilated cages (IVC). General anesthesia was induced and maintained by a 1–3% isoflurane/oxygen mix. Analgesia included carprofen (0.005 mg/kg s/c) and buprenorphine (0.01 mg/kg s/c) given at induction. Implantation of scaffold materials were performed through a 8 mm ‘off-midline’ dorsal incision and creating subcutaneous pockets in four quadrants over the dorsum. Scaffolds were implanted in two subcutaneous pockets and two muscle pockets. For muscle implantation, perimysium fascia was identified through a subcutaneous pocket and scaffolds implanted in the substance of the muscle. Animals were recovered and monitored daily. Postoperative analgesia (carprofen; 0.005 mg/kg s/c) was given for 24 h. Animals were terminated at 4 weeks or 8 weeks post implantation and scaffolds retrieved for analysis.

### 2.5 Statistical analysis

Data were analyzed using GraphPad Prism v6. ANOVA or Kruskal Wallis tests were applied as described in figure legends. *p*-values <0.05 were considered statistically significant.

## 3 Results

### 3.1 POSS-PCU polymer characterization after porogen dusting

#### 3.1.1 Mechanical properties

**Fig. 1:**
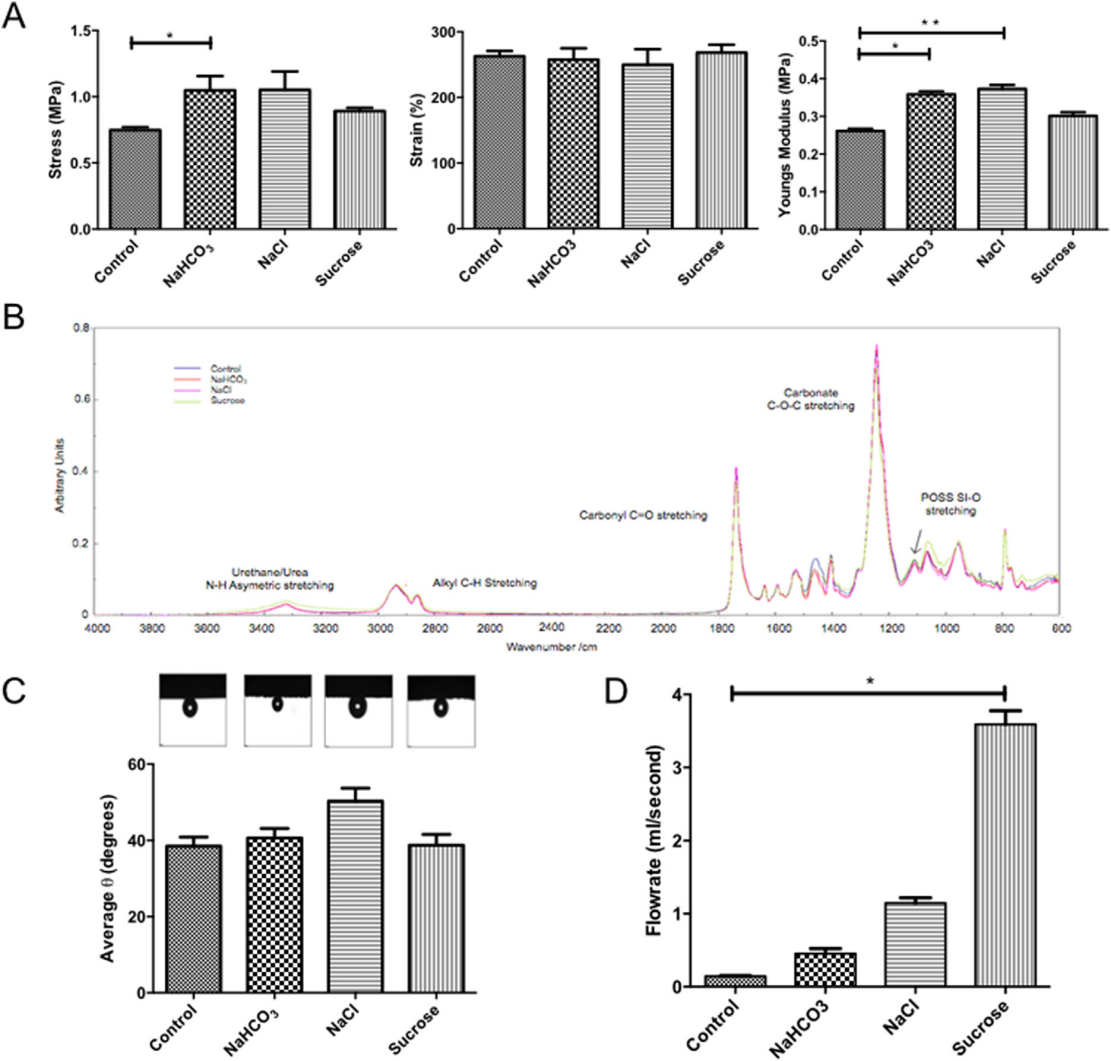
Characterization of POSS-PCU scaffolds of varying surface porosities. (A) Scaffolds were analyzed for their maximum stress, percentage strain and Young's modulus, which showed minimal changes between scaffolds. (B) ATR-FTIR spectroscopy demonstrated no changes between scaffolds. (C) Surface wetness via captive bubble contact angle also remained unchanged. (D) Permeability testing revealed the most significant changes following dusting with sucrose, which increased flow rate through the scaffold. Experiments were performed in triplicate and analyzed using a Kruskal–Wallis test with Dunn's correction for multiple comparisons. Statistical significance is displayed as *p < 0.05, **p < 0.01.

POSS-PCU scaffolds were dusted with three different sized porogens; NaHCO_3_, NaCl or sucrose. Mechanical properties were compared to non-dusted controls. A significant difference in maximum stress (p < 0.05) was only observed between the non-dusted control and NaHCO_3_-dusted samples (Fig. 1A). While no significant differences were observed in total strain (Fig. 1A), a significant increase in Young's modulus was observed between the non-dusted control and both the NaHCO_3_- and the NaCl-dusted samples (Fig. 1A).

#### 3.1.2 Surface chemistry

The surface chemistry of each sample was characterized using Attenuated Total Reflectance - Fourier Transform Infra-Red (ATR-FTIR) spectrometry. We observed the same spectra in all samples, with identical peaks showing absorption bands characteristic of poly(carbonate-urea-urethane). The largest absorptions occurred at 1737.55 cm^−1^ (carbonate C=O stretching from carbonate) and 1241.93 m^−^^1^ (carbonate C–O–C stretching; Fig. 1B). The absorption band for POSS is observed at 1111.76 cm^−1^, typical for POSS containing polyurethane (Fig. 1B). FTIR testing confirmed that the surface chemistry remained unaltered following fabrication. Absorption bands for all scaffolds were characteristic of poly(carbonate-urea) urethanes and confirmed that the addition of the various porogens did not change the overall chemistry of the surface.

#### 3.1.3 Contact angle

Despite a small increase in contact angle in the NaCl-dusted samples, there was no significant difference between dusted samples and non-dusted controls (Fig. 1C).

#### 3.1.4 Permeability

The rate at which water passes through the sample at a starting pressure of 40 mmHg was calculated. Increasing the porogen size used to dust the scaffolds increased the water flow rate through the scaffold. Therefore, the sucrose-dusted scaffold was the most permeable with an average flow rate of 3.59 ml/s (Fig. 1D).

#### 3.1.5 Scanning electron microscopy (SEM)

**Fig. 2:**
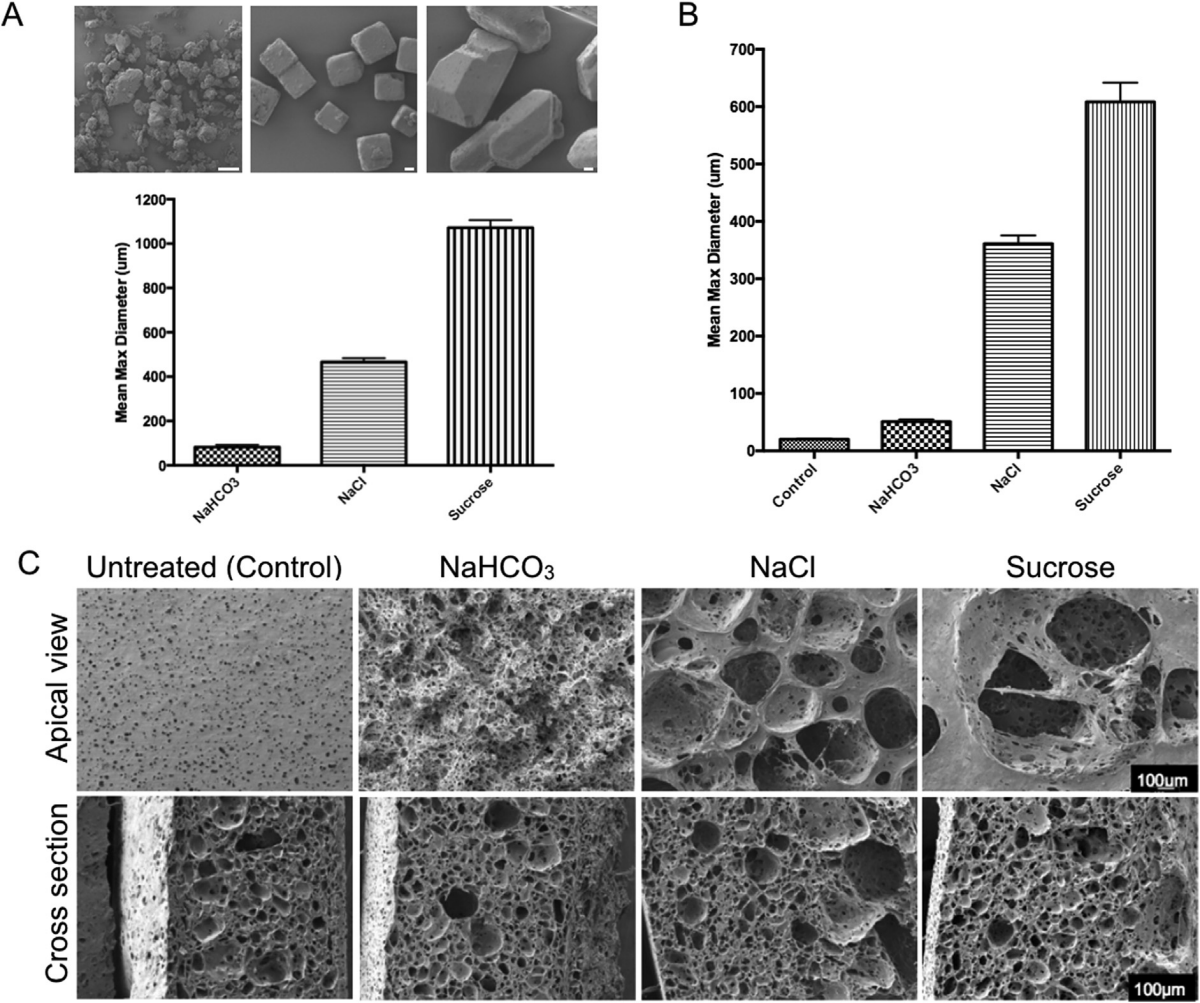
Surface topography of surface-dusted scaffolds. (A) Porogen size analysis using scanning electron microscopy (SEM) images and mean maximum diameter determined for each porogen type. (B) Analysis of pore size generated by each porogen in the scaffold (n = 50). (C) SEM images of the apical surface and a cross section of candidate scaffolds (scale bar = 100 μm).

SEM was used to quantify the average grain size of each porogen type and results showed that NaHCO_3_, NaCl and sucrose had increasing sizes respectively (Fig. 2A). SEM images of the top surfaces of dusted POSS-PCU scaffolds revealed that larger porogens produced larger pores (Fig. 2B) with marked differences in surface topography (upper panel; Fig. 2C). The inner material of the polymer remained equally porous across the different dustings as expected (lower panel; Fig. 2C).

### 3.2 Effects of POSS-PCU dusting on epithelial cytocompatibility

#### 3.2.1 Cell characterization

Human bronchial epithelial cells (HBECs) were derived from primary tissue and grown in submerged culture [30]. Staining with antibodies against cytokeratin 5 (CK5) and cytokeratin 14 (CK14) identified them as basal epithelial progenitor cells (Supplementary Figure 2A–C). Staining with an antibody against Ki67, a marker of cellular proliferation, showed the cells were actively proliferative on tissue culture plastic (Supplementary Figure 2D).

#### 3.2.2 Epithelial cell viability, growth, adherence and survival on POSS-PCU

**Fig. 3:**
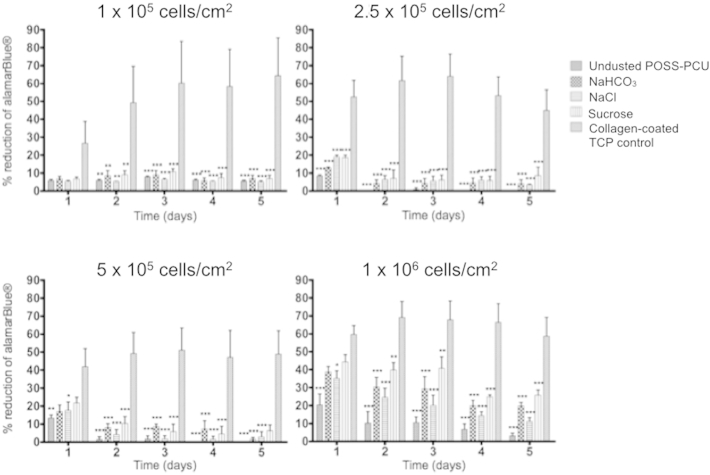
Human bronchial epithelial cell (HBEC) adherence, survival and growth on POSS-PCU. Metabolic activity of the cells on the four different scaffolds and the collagen-coated tissue culture plastic (TCP) control over 5 days are expressed as the percentage of alamarBlue reduced in the cells during the incubation. Experiments were performed in triplicate and analyzed using two-way ANOVA tests with Bonferroni post-tests. Statistical significance is displayed as *p < 0.05, **p < 0.01, ***p < 0.001.

Cell metabolic activity measurements using the alamarBlue assay showed that one day after seeding with four different cell densities, there were significantly fewer cells attached to the POSS-PCU scaffolds compared to a collagen-coated plastic control (Fig. 3). The metabolic activity of the cells on the control plastic increased up to day 3 and then reached a plateau at cell confluence. There were no significant differences in cellular metabolic activity between the dusted scaffolds at any time point across all seeding densities (Fig. 3). Of note, scaffolds seeded with 1 × 10^6^ cells/cm^2^ showed significantly higher metabolic activity on all the POSS-PCU scaffolds, indicating that large cell numbers are required for epithelial engraftment on these constructs (Fig. 3).

**Fig. 4:**
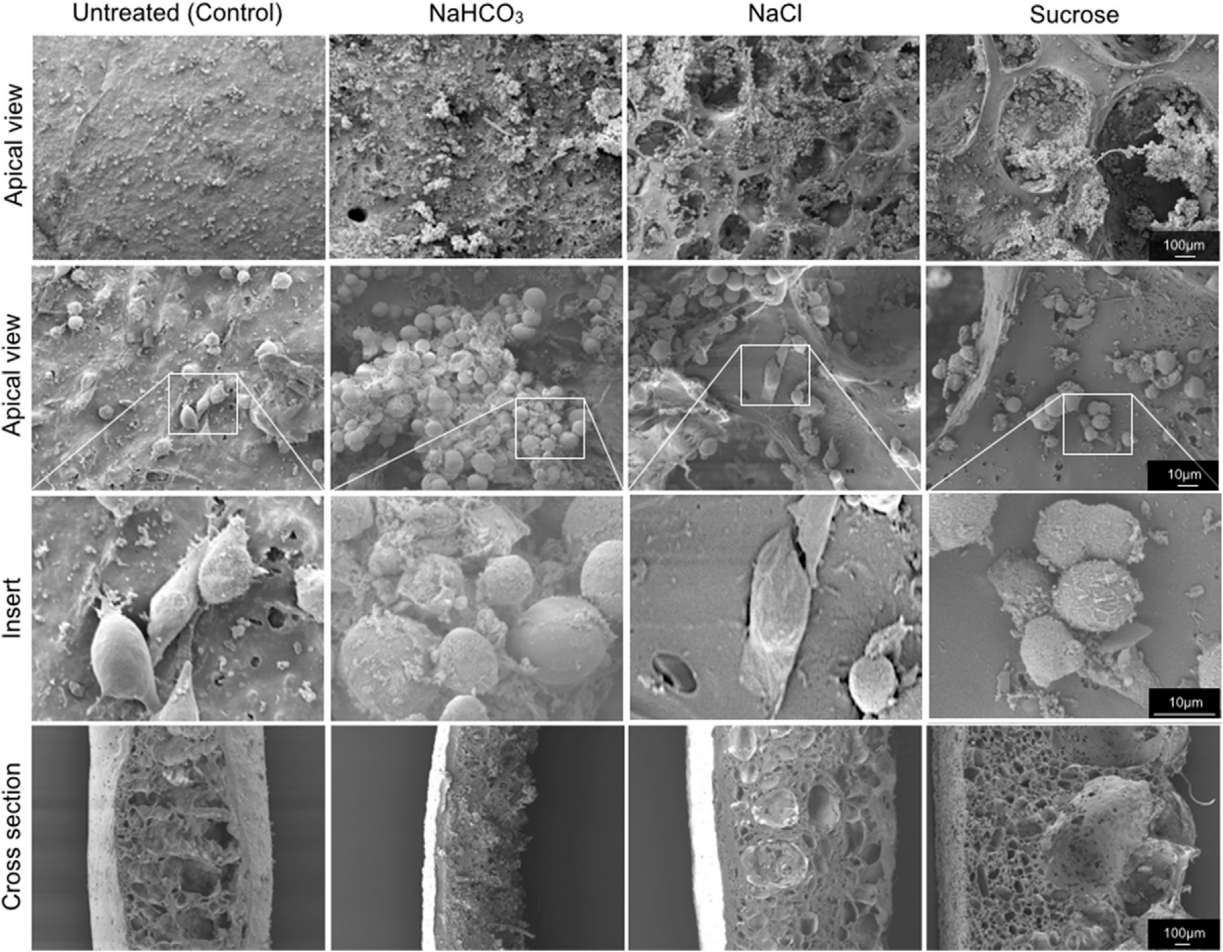
Human bronchial epithelial cells (HBECs) adhere but cluster on surface-modified POSS-PCU scaffolds. HBECs seeded on the scaffolds at 1 × 10^6^ cells/cm^2^ were glutaraldehyde-fixed two days post seeding and imaged by scanning electron microscopy. Top and middle rows show the dusted surface of the scaffolds at 75× and 400×, respectively. Third row shows magnified inserts of scanning electron microscope pictures demonstrating HBECs adhering to the scaffolds with evidence of early stages of cytoskeleton development, extrusion of podia and flattening. Bottom panel shows a cross section of the scaffolds at 75× magnification.

SEM images taken two days after seeding with 1 × 10^6^ cells/cm^2^ showed that the cells were evenly spread on the surface of the non-dusted and the NaHCO_3_-dusted scaffolds, which have small surface pores (Fig. 4). However, cells on the surface of NaCl and sucrose-dusted scaffolds had accumulated within the large pores (upper right panels; Fig. 4). Some cells appeared to weakly adhere to the scaffolds and showed early stages of cytoskeletal development and focal adhesion (Fig. 4). Notably, many of the cells within larger pores on the surface appeared to adhere to each other rather than to the scaffold surface, forming cellular aggregates. In all scaffolds except the non-dusted control, the cells appear to penetrate into the scaffolds through communicating pores (bottom panel; Fig. 4). Immunofluorescence staining for CK5 confirmed the clustering of cells within the pores (Supplementary Figure 3).

#### 3.2.3 Ethanol treatment improves cytocompatibility of non-dusted POSS-PCU scaffolds and allows epithelial differentiation

Having demonstrated adhesion of HBECs to POSS-PCU scaffolds, we attempted to culture cells for longer periods on non-dusted scaffolds but met limited success using autoclaved scaffolds. To address this issue we investigated whether the sterilization technique can affect the properties of POSS-PCU, finding that ethanol treatment produced scaffolds with a contact angle comparable to that of tissue culture plastic (Supplementary Figure 4A). Seeding with HBECs revealed that, while metabolic activity declined on autoclaved POSS-PCU over the course of two weeks of culture, it was retained on ethanol-treated scaffolds (Supplementary Figure 4B). Cells on ethanol-treated, non-dusted POSS-PCU retain an epithelial morphology (Supplementary Figure 4C). Given the ability of these scaffolds to maintain the viability of HBECs, we investigated whether cells could differentiate to form the functional cell types of the airway (mucosecretory goblet cells and ciliated cells) on POSS-PCU. After three weeks of culture at an air-liquid interface (ALI) cells formed a multi-layered epithelium with evidence of both secretory (MUC5B; Supplementary Figure 4D) and ciliated cell differentiation (ACT; Supplementary Figure 4D–4E).

### 3.3 Sucrose dusting of POSS-PCU leads to increased cellular infiltration and matrix deposition *in vivo*

**Fig. 5:**
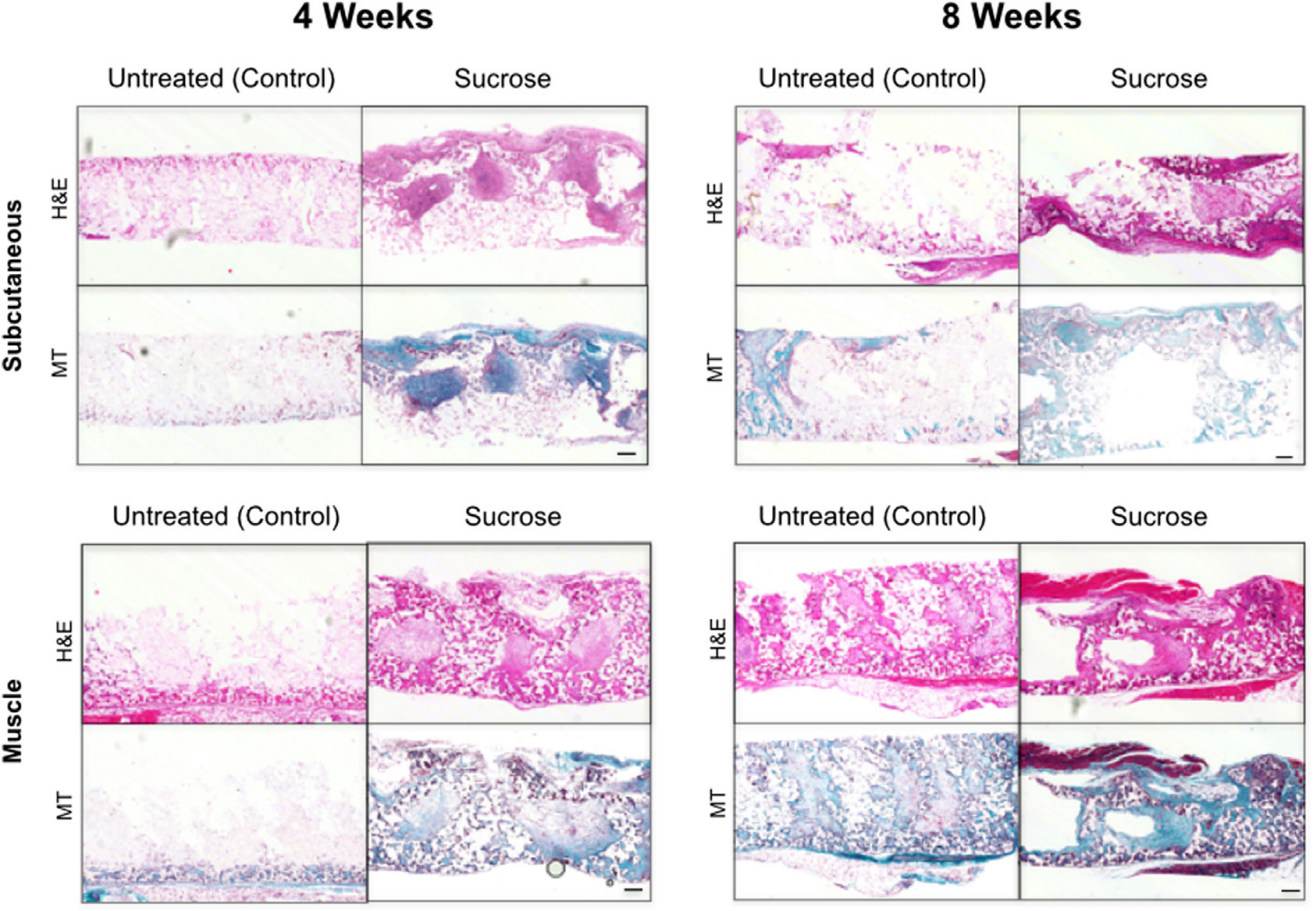
Integration of surface-modified POSS-PCU scaffolds after subcutaneous or intramuscular implantation. Histology images of implanted sucrose-dusted and non-dusted scaffolds at 4 weeks and 8 weeks post implantation. Scaffolds were either implanted subcutaneously or into muscle. Staining with haematoxylin and eosin (H&E) demonstrates cell infiltration into scaffolds and Masson's trichrome [22] shows matrix deposition. These highlight a significant cell infiltrate and matrix deposition in sucrose-dusted scaffolds that have been implanted in muscle at both early and late time points and subcutaneously at the later 8 week time point (scale bar = 100 μm).

**Fig. 6:**
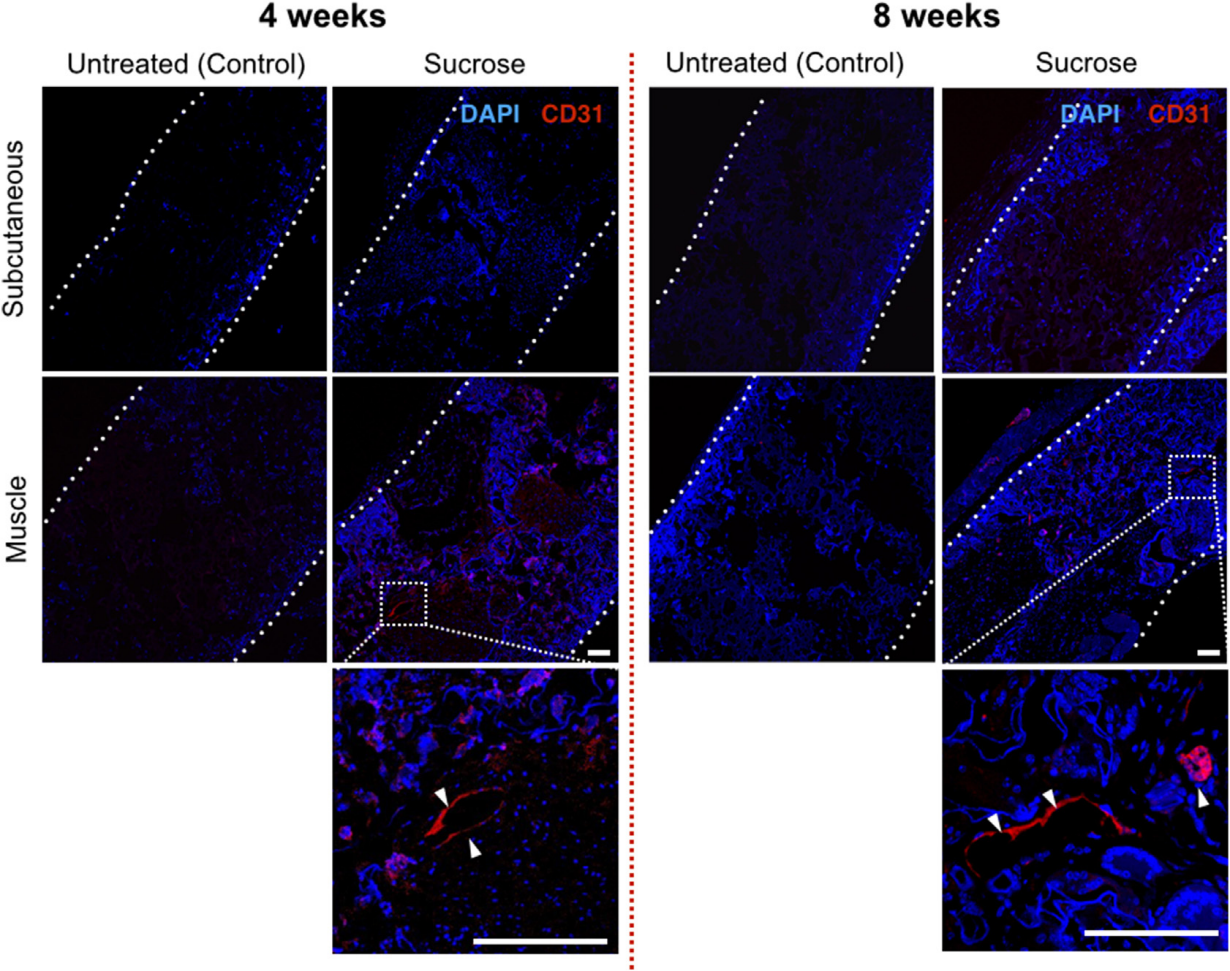
Sucrose dusting of POSS-PCU increases neo-vascularization *in vivo*. Immunofluorescence staining of implanted sucrose-dusted and non-dusted samples at 4 weeks and 8 weeks post implantation. Scaffold edges are highlighted by dotted lines. DAPI nuclear counter stain shows minimal cell infiltrate in untreated scaffolds compared to sucrose-dusted scaffolds, where cells are present throughout the graft. CD31 staining (white arrows) confirms the presence of vessels within the sucrose-dusted scaffold (scale bar = 100 μm).

Integration of scaffolds within a host tissue is a huge challenge in synthetic polymer bioengineering [31] so we examined the integration of surface-modified POSS-PCU *in vivo*. Scaffolds can be implanted as a free graft or on a pedicled vascular flap to improve revascularization, so discs were implanted either subcutaneously or into the dorsal muscle of C57BL/6 immunocompetent mice and harvested after 4 or 8 weeks. We compared non-dusted scaffolds to sucrose-dusted scaffolds given that these had the largest pore size and permeability of our surface-modified scaffolds. All animals survived with no adverse reactions to the grafts. On macroscopic inspection, sucrose-dusted samples appeared better integrated than non-dusted samples. Haematoxylin and eosin (H&E) staining confirmed enhanced cellular integration in the sucrose-dusted scaffolds compared to the non-dusted samples (Fig. 5). Further, Masson's trichrome staining demonstrated matrix deposition within the pores, which was more evident in the muscle-implanted samples compared to those implanted subcutaneously (Fig. 5). H&E staining also revealed differences in the time taken to recellularize the graft with an advantage conferred by muscle implantation. Individual pores had increased numbers of cells in the sucrose-treated group compared to non-dusted scaffolds (Fig. 5). Immunofluorescence for the nuclear marker DAPI demonstrated increased nuclear counts in the center of the graft, suggesting improved cell ingrowth in scaffolds with larger pores (Fig. 6). Staining for the endothelial cell marker CD31 confirmed evidence of increased neovascularization within the sucrose-dusted scaffolds (Fig. 6).

**Fig. 7:**
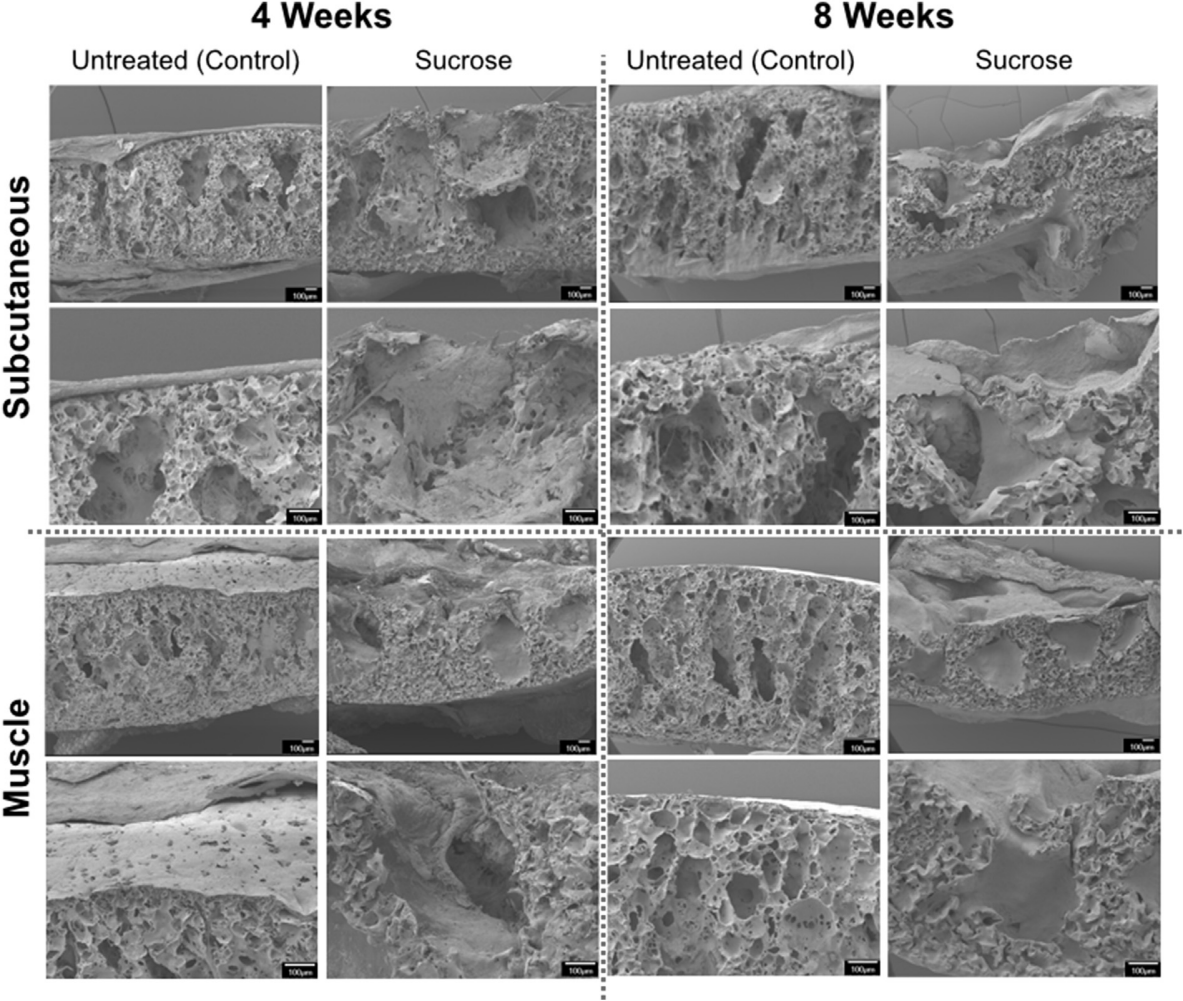
Integration of POSS-PCU is improved by increased surface porosity conferred by surface dusting. Scanning electron microscopy (SEM) images of implanted dusted and non-dusted POSS-PCU scaffolds at 4 and 8 weeks post implantation in cross section. Non-dusted scaffolds consistently demonstrated a capsule-like structure with minimal integration compared to sucrose-dusted POSS-PCU scaffolds, which showed tissue ingrowth into individual pores.

SEM images provide insight into the effect of dusting POSS-PCU dusting on cell ingrowth *in vivo*. Non-dusted samples had a sheet-like capsule structure surrounding the scaffold surface, suggesting that small pores were a barrier to integration. On the other hand, dusted samples demonstrated matrix deposition within the scaffold pores (Fig. 7) consistent with our data that these integrated more readily with host tissue *in vivo*.

## 4 Discussion

Various methods of creating pores within materials exist, including gas forming, phase separation, freeze-drying and salt leeching, described in detail by Lon et al. [32]. Here, we describe methods for altering surface topography and porosity of POSS-PCU using three different porogens, NaHCO_3_, NaCl and sucrose, and characterize the effects of these modifications on material properties, cytocompatibility and *in vivo* response.

The POSS-PCU constructs used for hollow organ design have aimed principally to maintain barrier function, an application for which a closed (minimal) pore design is logically best suited. This creates a uniform surface but, as a result, the potential oxygen gradient between the luminal surface and the external surface of the graft may be large, exposing luminal cells to hypoxic conditions and poor gas exchange [33]. Determining the optimal scaffold porosity is essential for the delivery of metabolites and to create an appropriate microenvironment for vascular ingrowth. Open, interconnecting pores could provide mechanical support but at the same time act as a physical stimulus to guide and promote tissue and vascular regeneration [32]. Additionally, pores on the surface of the scaffold might facilitate interlocking between the scaffold and surrounding tissues, thereby providing increased overall mechanical stability to the implant [34]. Design of a scaffold that can provide this stability combined with adequate delivery of metabolites and maintained luminal barrier function will be important for improving clinical delivery of synthetic scaffolds.

Importantly, the introduction of pores into POSS-PCU through dusting did not lead to diminished structural integrity. These data are reassuring but mechanical differences may become apparent when scaffolds are up-scaled to tissue/organ-specific sizes. Since POSS-PCU constructs can be manufactured as composites consisting of both the solid- and the foam-form elastomers, such deficiencies could be readily accommodated by scaffold design. This is particularly relevant for the trachea where the solid-form of POSS-PCU provides the mechanical strength in the ‘c’-shaped rings and the foam-form provides the softer interconnecting struts to produce a biomimetic scaffold reminiscent of native tissue [15].

We confirmed by FTIR and contact angle analysis that modifications would not alter POSS-PCU chemistry or surface wetness. We measured permeability as a surrogate measure of interconnecting pores, which would ultimately be necessary for tissue ingrowth. Untreated POSS-PCU has a small surface pore size of approximately 20 μm. Dusting opened these pores and permeability tests showed treatment with sucrose, the largest porogen, improved porosity more than either NaHCO_3_ or NaCl. There is evidence that capillary ingrowth into biomaterials is most encouraged with pores of between 40 μm and 600 μm [35] and here we generated pores of approximately 600 μm (sucrose treated) 350 μm (NaCl-treated) and 50 μm (NaHCO_3_-treated) (Fig. 2). The penetration of the porogens into the scaffold is dependent on a number of factors. These include the porogen type, the amount of porogen used and the duration of time between dusting the polymer and its immersion in water. Therefore, the depth of the pores can be controlled. In this study, we immediately placed the dishes in water following dusting to allow limited time for the porogens to sink into the polymer. Our ongoing and future research aims to determine the optimum penetration of the porogen and to validate this within our protocol.

Given the necessity for barrier function in tubular constructs [36], we determined whether porogen modifications would be detrimental to epithelial engraftment. POSS-PCU was broadly cytocompatible, with or without dusting modifications. Ethanol sterilization of non-dusted POSS-PCU scaffolds allowed improved epithelialization and large pore size also appears to have improved epithelial cell attachment *in vitro*. However, this latter result may be explained by cell aggregation within pores, rather than by cohesive engraftment as an epithelial sheet. Dusting appears poorly suited to *ex vivo* epithelialization due to disruption of the flat luminal surface. However, current untreated synthetic scaffolds will also require significant improvements to promote epithelial attachment [37]. Future modifications could include functionalizing the scaffold with peptides [35], growth factors [38] or antibodies [39], [40]; plasma-enhanced chemical vapor deposition techniques [41]; addition of stromal and/or endothelial cells [42]; and chemical/biochemical patterning of the scaffold surface via microcontact printing [43]. Current work is in progress to enhance the surface properties using such methodologies as well as to 3D print scaffolds with defined topographical structures [44], [45].

Importantly, we demonstrated that sucrose dusting and the consequent generation of interconnecting pores increased cell ingrowth *in vivo*. Even at eight weeks post transplantation, non-dusted scaffolds had not integrated well with host tissue. Interestingly, dusted scaffolds implanted in muscle showed tissue ingrowth, neoangiogenesis and matrix deposition much earlier than those implanted subcutaneously. This suggests the importance of implantation into a well-vascularized site for rapid integration and will guide studies assessing the period of time that full-sized scaffolds might require prior to clinical orthotopic transplantation.

Overall, our results indicate that, while future modifications of the apical surface of POSS-PCU will be necessary in organs with a barrier function, dusting of POSS-PCU scaffolds may significantly improve their integration into host tissue.

## 5 Conclusion

The methods described here allow the controlled modification of the surface of POSS-PCU scaffolds. Porogen dusting of POSS-PCU increases graft integration with surrounding host tissues, including vascular ingrowth. The ability to manipulate porosity represents a sizable advancement for POSS-PCU and increases the scope of current and future POSS-PCU applications.
