# Supplementary material for: Surface modification of a POSS-nanocomposite material to enhance cellular integration of a synthetic bioscaffold
Source: Biomaterials. 2016 Mar;83:283–93. doi: 10.1016/j.biomaterials.2016.01.005 (PMC4762251; doi:10.1016/j.biomaterials.2016.01.005)
Supplement: Supplementary file 1 [file mmc1.docx]

**Supplementary Data**

**Surface modification of a POSS-nanocomposite material to enhance cellular integration of a synthetic bioscaffold**

Claire Crowleya,b, Poramate Klanritb, Colin R. Butlera, Aikaterini Varanoua, Manuela Platéa,c, Robert E. Hyndsa, Rachel C. Chambersc, Alexander M. Seifalianb, Martin A. Birchalld and Sam M. Janesa

a Lungs for Living Research Centre, UCL Respiratory, University College London, London, UK

b UCL Centre of Nanotechnology and Regenerative Medicine, Division of Surgery and Interventional Science, Royal Free London NHS Foundation Trust Hospital and University College London, London, UK

c Centre for Inflammation and Tissue Repair, UCL Respiratory, University College London, London, UK

d UCL Ear Institute, Royal National Throat Nose and Ear Hospital and University College London, London, UK

**Authors for correspondence:**

Professor Sam M. Janes

Lungs for Living Research Centre, UCL Respiratory, University College London, 5 University Street, London WC1E 6JF, United Kingdom

Tel: +44 (0) 207 679 6926 Email: [s.janes@ucl.ac.uk](mailto:s.janes@ucl.ac.uk)

Professor Martin A. Birchall

Royal National Throat, Nose and Ear Hospital, 330 Gray’s Inn Road, London, WC1X 8DA, United Kingdom

Tel: +44 (0) 203 456 5007 Email: [m.birchall@ucl.ac.uk](mailto:m.birchall@ucl.ac.uk)

**
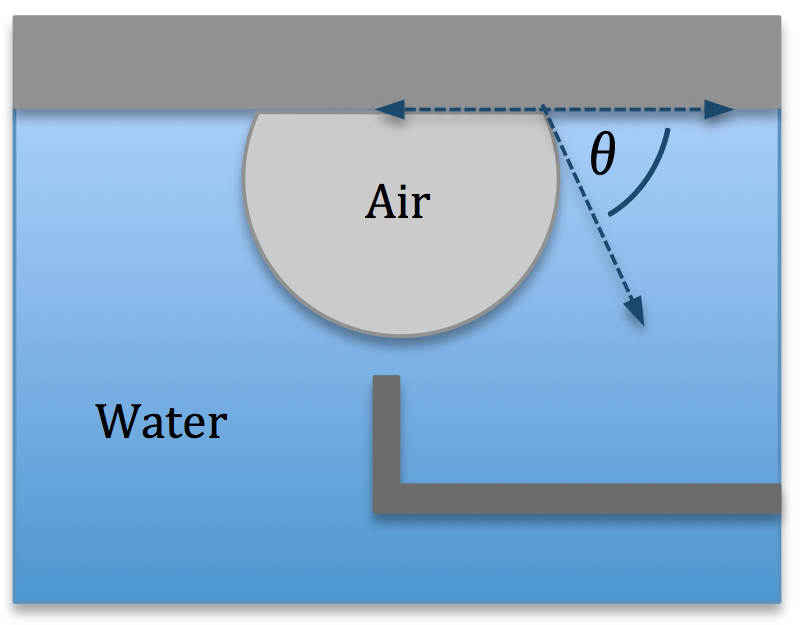
**

**Supplementary Figure 1: Schematic representation of the captive bubble method used to analyze the wettability of POSS-PCU surfaces.**

Due to the porous nature of the surface of the scaffolds, the captive bubble method for drop application was used to determine the wettability of the surface. An air bubble is applied to the surface of the scaffold and the angle at which the bubble interacts with the scaffold is calculated. Contact angle units are given in degrees θ.


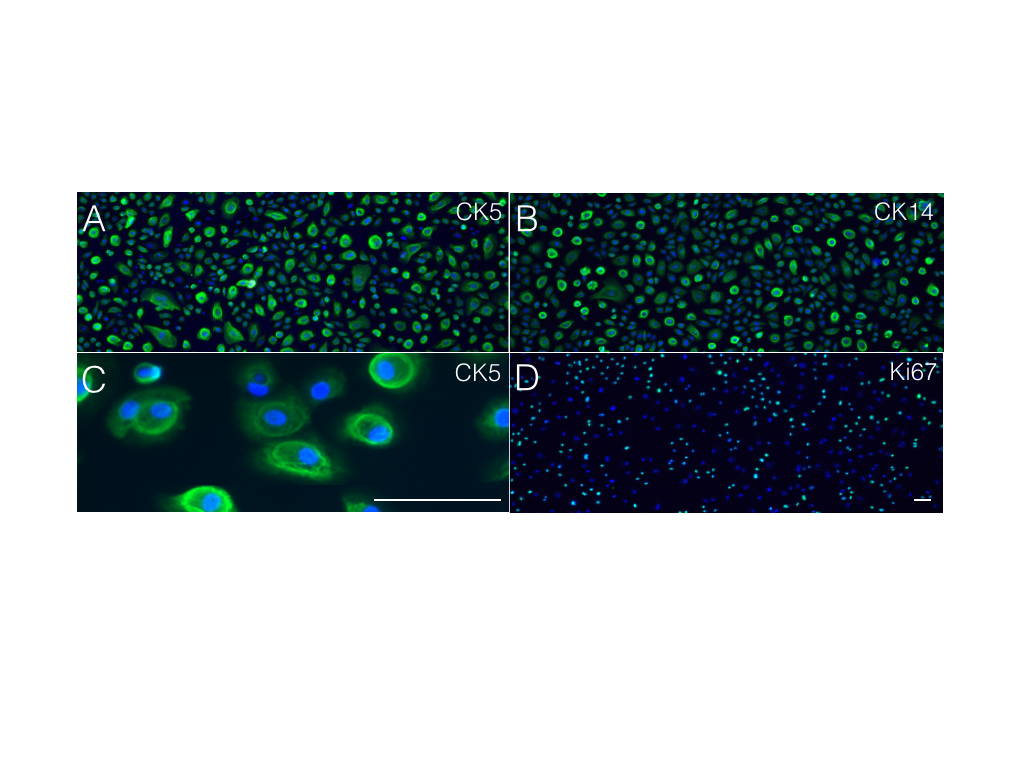


**Supplementary Figure 2: Basal epithelial cell characteristics of primary** **human bronchial epithelial cells (HBECs).**

Human bronchial epithelial cells (HBECs) grown in submerged culture were stained for CK14 and CK5 (A-C) and Ki67 (D). As anticipated, isolated respiratory epithelial cells expressed basal progenitor markers and were proliferative (scale bars = 50 μm).


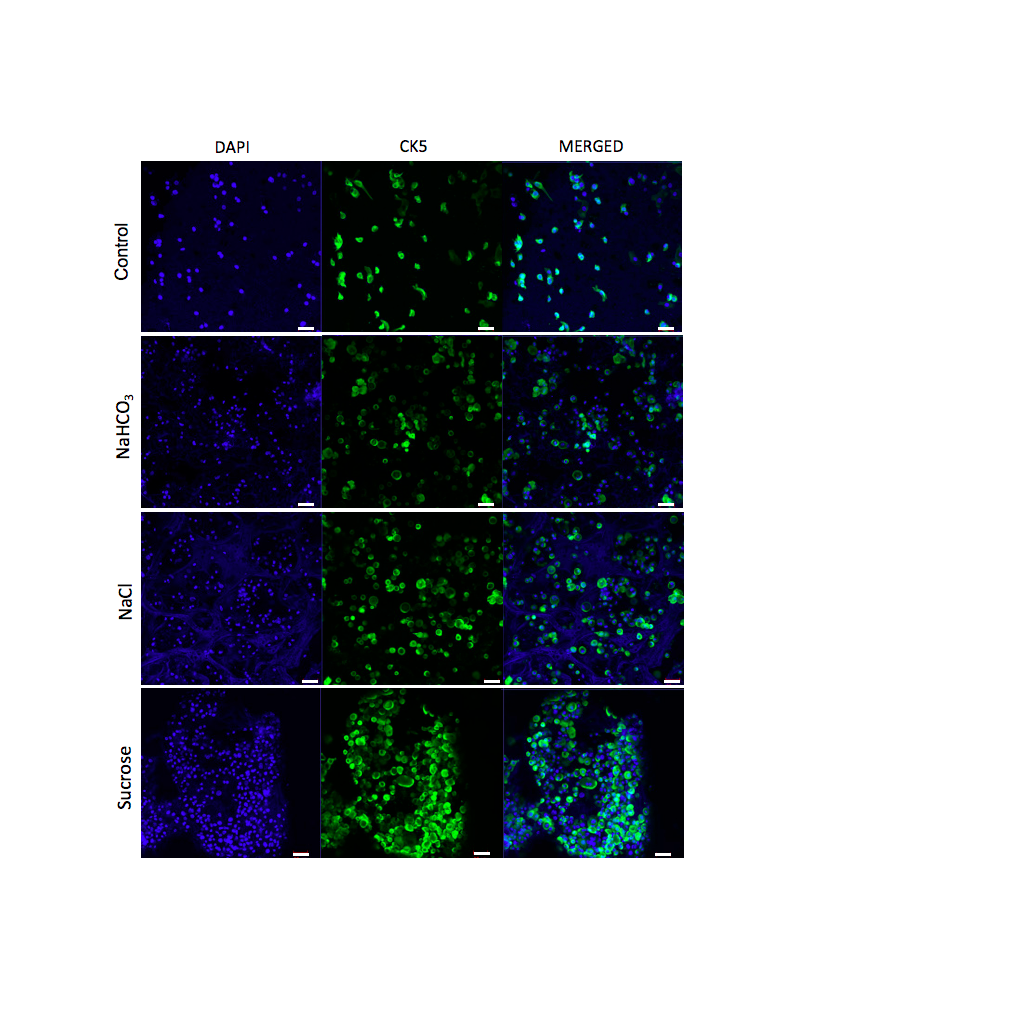


**Supplementary Figure 3: Immunofluorescence staining of human bronchial epithelial cells (HBECs) seeded on POSS-PCU scaffolds.**

Human bronchial epithelial cells (HBECs) seeded on the different types of scaffolds at 1 x 10^6^ cells/cm^2^ were PFA-fixed at two days after seeding and immunofluorescence stained for CK5 (green), a marker of basal epithelial cells. Nuclei were counterstained with DAPI (blue). Z-stack images were acquired by confocal microscopy and an extended focus merged image generated. This confirmed increased clustering of epithelial cells in scaffolds with larger pores.


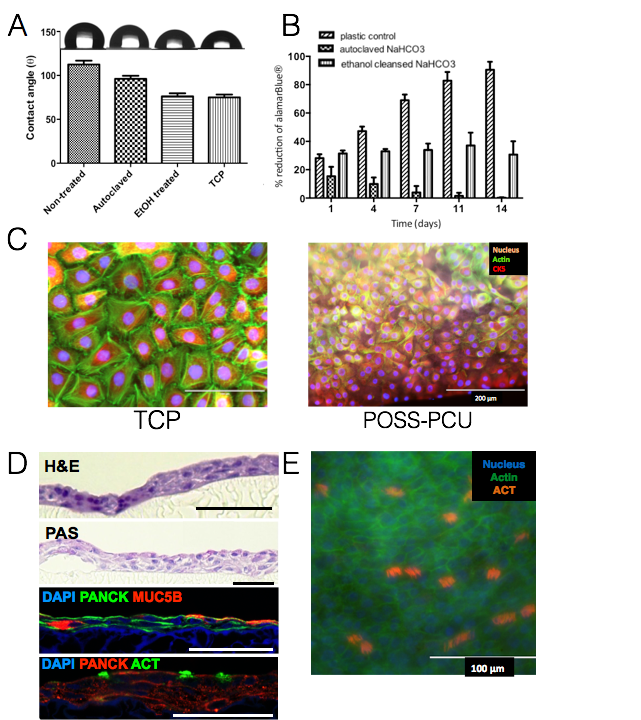


**Supplementary Figure 4: Ethanol sterilization maintains human bronchial epithelial cell (HBEC) viability and allows differentiation on unmodified POSS-PCU.**

(A) Comparison of contact angle in scaffolds sterilized by either autoclaving or ethanol treatment. (B) Analysis of metabolic activity using alamarBlue assay on tissue culture plastic, autoclaved and ethanol-treated POSS-PCU scaffolds. (C) Primary human bronchial epithelial cell (HBEC) morphology on tissue culture plastic and non-dusted POSS-PCU scaffold. (D) Histological analysis of HBECs cultured on non-dusted POSS-PCU scaffolds for 14 days. (E) Top down confocal microscopy of epithelial sheet surface after 14 days of culture shows ciliated differentiation (ACT; red).
